# *Hovenia dulcis* Fruit Peduncle Polysaccharides Reduce Intestinal Dysbiosis and Hepatic Fatty Acid Metabolism Disorders in Alcohol-Exposed Mice

**DOI:** 10.3390/foods13081145

**Published:** 2024-04-09

**Authors:** Liangyu Liu, Sijie Zhu, Yuchao Zhang, Zhenyuan Zhu, Yong Xue, Xudong Liu

**Affiliations:** 1 College of Food Science and Engineering, Ocean University of China, Qingdao 266003, China; aleliuliangyu@163.com; 2 Department of Food Science and Engineering, Moutai Institute, Renhuai 564507, China; 18798006458@163.com; 3 College of Food Science and Engineering, Tianjin University of Science and Technology, Tianjin 300222, China; zhyuanzhu@tust.edu.cn; 4 Department of Brewing Engineering, Moutai Institute, Renhuai 564507, China; zyc10271027@163.com

**Keywords:** *Hovenia dulcis* fruit peduncle polysaccharides, alcohol abuse, dyslipidemia, intestinal dysbiosis, hepatic fatty acid metabolism disorders

## Abstract

Alcohol abuse can lead to alcoholic liver disease, becoming a major global burden. *Hovenia dulcis* fruit peduncle polysaccharides (HDPs) have the potential to alleviate alcoholic liver injury and play essential roles in treating alcohol-exposed liver disease; however, the hepatoprotective effects and mechanisms remain elusive. In this study, we investigated the hepatoprotective effects of HDPs and their potential mechanisms in alcohol-exposed mice through liver metabolomics and gut microbiome. The results found that HDPs reduced medium-dose alcohol-caused dyslipidemia (significantly elevated T-CHO, TG, LDL-C), elevated liver glycogen levels, and inhibited intestinal-hepatic inflammation (significantly decreased IL-4, IFN-γ and TNF-α), consequently reversing hepatic pathological changes. When applying gut microbiome analysis, HDPs showed significant decreases in Proteobacteria, significant increases in Firmicutes at the phylum level, increased *Lactobacillus* abundance, and decreased *Enterobacteria* abundance, maintaining the composition of gut microbiota. Further hepatic metabolomics analysis revealed that HDPs had a regulatory effect on hepatic fatty acid metabolism, by increasing the major metabolic pathways including arachidonic acid and glycerophospholipid metabolism, and identified two important metabolites—C00157 (phosphatidylcholine, a glycerophospholipid plays a central role in energy production) and C04230 (1-Acyl-sn-glycero-3-phosphocholine, a lysophospholipid involved in the breakdown of phospholipids)—involved in the above metabolism. Overall, HDPs reduced intestinal dysbiosis and hepatic fatty acid metabolism disorders in alcohol-exposed mice, suggesting that HDPs have a beneficial effect on alleviating alcohol-induced hepatic metabolic disorders.

## 1. Introduction

As stated by the World Health Organization (WHO), about 3.1 billion people over the age of 15 are drinkers, and per capita consumption of alcohol can reach 32.8 g/day, exceeding the “Dietary Guidelines” for daily drinking (Chinese men or women should not consume more than 25 or 15 g/day, American men or women should not consume more than 28 or 15 g/day) [1,2]. Alcohol abuse is a global problem and is widely recognized as one of the major causes of liver disease. Both short-term acute binge drinking and long-term alcohol abuse can cause serious damage to the liver, including fatty liver, alcoholic hepatitis, liver cirrhosis, and carcinoma [3]. Alcohol is mainly absorbed through the gastrointestinal tract, and alcohol-induced dysregulation of intestinal microecology and consequent disruption of hepatic lipid metabolism has been extensively studied [4,5]. Studies have suggested that chronic alcohol abuse interferes with the intestinal microecological balance, leading to dysbiosis of the microbial community; this microecological disturbance may affect the intestinal barrier function and increase the infiltration of endotoxins, thus triggering inflammatory responses and abnormalities in hepatic lipid metabolism [6,7]. In addition, alcohol abuse alters the composition and function of intestinal microorganisms, leading to disorders of hepatic lipid metabolism and increased hepatic fats deposition; the accumulation of these fats in the liver may lead to the development of fatty liver and other liver diseases [8]. Alcohol intake can also lead to hepatocellular damage and oxidative stress, further exacerbating hepatic lipid metabolism disorders [9]. Other studies have shown that the alcohol metabolite acetaldehyde inhibits key enzyme activities in lipid metabolic pathways and interferes with the oxidative metabolism of fatty acids; such metabolic disturbances may lead to aberrant accumulation of lipids and abnormalities in lipid metabolism [10]. Overall, alcohol induces intestinal microecological disturbances and indirectly contributes to hepatic lipid metabolism disorders, which may be one of the mechanisms of alcohol damage to the liver [7,11]. However, with alcohol known to be indispensable in life, we still need to understand how it affects intestinal microecology and hepatic lipid metabolism [12]. To prevent and treat liver diseases caused by alcohol, it is important to search for natural products with hepatoprotective effects as well as conduct further research into the mechanisms of such protective effects.

*Hovenia dulcis* (known in China as Guaizao and in Japan as Japanese grape) is a traditional medicinal and edible plant in China, Korea, and Japan, and is a member of the Rhamnaceae family. As a traditional herbal remedy for liver disease and alcohol toxicity, *Hovenia dulcis* has been the subject of extensive research [13]. A previous study has indicated that both the juice and fermented vinegar from *Hovenia dulcis* peduncles offer a protective benefit against the biochemical alterations induced by long-term ethanol consumption in mice [14]. Moreover, a controlled trial, which was randomized, double-blind, and placebo-controlled, suggested that the extract from *Hovenia dulcis* fruit could help alleviate hangover symptoms and mitigate liver damage caused by alcohol in humans [15]. Recent investigations have revealed that the active ingredients found in *Hovenia dulcis* possess antioxidant, anti-inflammatory, and anti-fibrotic properties, which can inhibit harmful substances produced during the metabolism of alcohol and reduce damage to the liver [16,17,18]. Despite this, the majority of research has concentrated on the seeds of *Hovenia dulcis*, with less attention given to the fruit peduncle. The *Hovenia dulcis* fruit peduncle (edible fleshy part) accounts for 90% of the total fruit, which is rich in a variety of bioactive substances, mainly polysaccharides, and offers significant developmental advantages and health benefits [19,20,21]. As the main bioactive substance in *Hovenia dulcis* fruit peduncle, *Hovenia dulcis* fruit peduncle polysaccharides (HDPs) have been proven to be effective in restoring the upright reflex of drinking mice, shortening their sleep time and coma state [22]. Other studies suggest that acute/chronic alcoholic liver disease damage, non-alcoholic fatty liver injury, and dyslipidemia can also be regulated by HDPs through anti-inflammatory, anti-lipid peroxidation and regulation of intestinal permeability [23,24]. In our recent study, we extracted HDPs and confirmed their capacity to efficiently neutralize free radicals as well as to prevent the oxidation of biomolecules (proteins, lipids, and DNA) in vitro [25]. Although there has been an increase in research on *Hovenia dulcis* and its various health benefits, particularly in alleviating symptoms of liver disease and alcohol poisoning, a significant research gap exists regarding the comprehensive understanding of the mechanisms by which HDPs exert their therapeutic effects. While the antioxidant, anti-inflammatory, and anti-fibrotic properties of HDPs have been established, the detailed pathways through which they modulate hepatic lipid metabolism and mitigate alcohol-induced liver damage are not yet fully elucidated. The innovative aspect of our research is the exploration of the role of HDPs in regulating hepatic lipid metabolism disorders, an area that has been largely uncharted. 

Therefore, this present study takes the HDPs obtained from *Hovenia dulcis* fruit peduncle as the object, aiming to explore the protective effects of HDPs on alcohol-induced acute liver injury, and applying intestinal 16S rRNA gene sequencing analysis combined with hepatic metabolomics to gain insights into its possible mechanisms. This study provides a theoretical basis for the application of HDPs in functional food and further reveals the mechanism of alcoholic liver injury.

## 2. Materials and Methods

### 2.1. Extraction and Characterization of HDPs

HDPs were obtained using the extraction method from Yang et al. [21] and our previous study [25]. Briefly, the pretreatment *Hovenia dulcis* fruit peduncle was initially degreased, and crude HDPs was prepared by water extraction and ethanol precipitation under the following conditions: a solid–liquid ratio of 1:25 g/mL, an extraction temperature of 85 °C, an extraction time of 1 h, and an ethanol precipitation volume fraction of 80%. Then, crude HDPs were deproteinized through Sevage reagent, followed by decolorization with AB-8 macroporous resin. Subsequently, the sample was subjected to dialysis using a membrane with a molecular weight cut-off of 8000 to 14,000 Da. This was followed by a 48 h dialysis against tap water and an additional 24 h dialysis against distilled water, with the water being refreshed every 12 h. The final step involved vacuum freeze-drying to yield the HDPs.

The molecular morphologies of polysaccharides were observed using a scanning electronic microscope (Zeiss Merlin Compact, Oberkochen, Germany) with magnification 2000×. Prior to measurements, the specimens’ surfaces were coated with a thin gold film to optimize conductivity. The sample was uniformly adhered to the sample stage and then scanned at a volte of 1.0 KV in vacuum [26]. The molecular weight and conformation of the HDPs were assessed using a Size Exclusion Chromatography system equipped with Multi-Angle Light Scattering and Refractive Index Detection (SEC-MALLS-RI). The system comprised a U3000 liquid phase (Thermo Fisher, Waltham, MA, USA), an Optilab T-rEX differential oscillometric detector (Wyatt technology, Santa Barbara, CA, USA), and a DAWN HELEOS II laser light scattering detector (Wyatt technology, Santa Barbara, CA, USA). A series of gel exclusion chromatography columns (OHpak SB-805 HQ, OHpak SB-804 HQ, and OHpak SB-803 HQ, each 300 × 8 mm) were utilized in tandem [27]. The Fourier transform infrared (FT-IR) spectra of the HDPs were captured with a spectrometer (Nicolet iZ-10, Thermo Nicolet, Waltham, MA, USA). For this, the HDPs were blended with KBr powder and formed into 1 mm pellets for FT-IR analysis in the wavenumber range of 4000 to 400 cm^−1^ [28]. Additionally, the monosaccharide composition of the HDPs was determined through high-performance anion-exchange chromatography (HPAEC, ICS-5000+, Thermo Fisher, Sunnyvale, CA, USA) on a CarboPac PA-20 anion-exchange column (3 by 150 mm, Dionex, Germering, Germany), employing a pulsed amperometric detector (PAD, ICS 5000 system, Dionex, Germering, Germany) [27].

### 2.2. Animal Exposure

Male C57BL/6 mice (weighing about 20 g, 6 weeks old, No. 430727221102531037) were purchased from Hunan Animal Experimentation Center, and housed in a standard environment with constant temperature (20 ± 2) °C, constant humidity (50 ± 2)%, and light/darkness for 12 h. Mice were acclimatized for 3 days prior to the experiment. Eighty mice were randomly divided into four groups (n = 20): control (CON) group, Low dose of alcohol (Low_ALC), Medium dose of alcohol (Medium_ALC) and HDPs + Medium dose of alcohol (HDPs_ALC). According to the per capita consumption of alcohol (32.8 g/day), the alcohol exposure dose of mice was 114 μL/20 g after a dose equivalent conversion for humans and mice [29]. Considering that the health effects of low dose alcohol intake are still of great concern, the study also chose 11.4 μL/20 g as a low exposure dose. Equal amounts of distilled water were administered to the control group by gavage at regular intervals every day, and 11.4 μL/20 g and 114 μL/20 g of 95% edible alcohol (CAS: 20220727-Z703, production standard GB31640-2016) were administered to the mice in the Low_ALC and Medium_ALC groups at regular intervals every day, respectively. The HDPs_ALC group was administered first by gavage with 100 mg/kg HDPs for 2 h [21], and then 114 μL/20 g of 95% edible alcohol was administered by gavage. The treatments were continuous for 15 days. A commercial diet and filtered water were provided ad libitum. All animal studies were conducted according to the protocol approved by the Animal Care and Use Committee of Moutai Institute (No. MTI-IACUC-2022-007).

### 2.3. Serum Biochemical Analysis

The blood of the mice sacrificed was centrifuged at 2000 r/min for 10 min at 4 °C, and the serum was collected. Using total cholesterol (TC, CAS: A111-1-1), triglyceride (TG, CAS: A110-1-1), low-density lipoprotein cholesterol (LDL-C, CAS: A113-1-1), and high-density lipoprotein cholesterol (HDL-C, CAS: A112-1-1) assay kits purchased from Nanjing Jiancheng Co., serum TC, TG, LDL-C and HDL-C levels were measured. The serum activities for alanine aminotransferase (ALT, CAS: C009-2-1) and aspartate aminotransferase (AST, CAS: C010-2-1) were detected using commercial assay kits (Nanjing Jiancheng Co., Nanjing, China).

### 2.4. Histological Examination

The fresh liver tissues of mice from each group were preserved in 4% paraformaldehyde solution for 24 h, routinely dehydrated, and embedded in paraffin, with a section thickness of 5 μm for hematoxylin-eosin (H&E) staining. Also, acid–Schiff staining (PAS) of the deparaffined sections was conducted (CAS: G1281, Solarbio, Beijing, China) to observe glycogen changes in liver tissues [30]. These sections were digitized using a digital slide scanner (Panoramic MIDI, 3DHISTECH, Budapest, Hungary) and subsequently observed using y the CaseViewer software (3DHISTECH, Budapest, Hungary) [31].

### 2.5. Biological Determination of Injury-Related Parameters in Liver and Intestine

Liver and intestine tissues of mice were collected to detect the levels of interleukin-4 (IL-4, CAS: H005-1-2), interferon-γ (IFN-γ, CAS: H025-1-2) and tumor necrosis factor-α (TNF-α, CAS: H052-1-2) using ELISA commercial assay kits (Nanjing Jiancheng Co., Nanjing, China). Glycogen content was detected in the liver, and the levels for lipase (LPS, CAS: A054-2-1), lipopolysaccharide binding protein (LBP, CAS: A043-1-1), and α-amylase (AMS, CAS: C016-1-1) were detected in the intestine. Protein concentrations of the sample were measured with BCA protein analysis kit (Beyotime, Shanghai, China). 

### 2.6. Gut Microbiota Analysis

Intestines (n = 4 repeats, 3 intestine samples pooled as one repeat) were used for 16S rRNA gene amplicons sequencing. Briefly, genomic DNA was extracted from 50 mg of tissue samples and detected through 2% agarose gel electrophoresis, amplified using an ABI GeneAmp^®^ 9700 PCR (ABI, Los Angeles, CA, USA), and the primer is 338F-806R. The PCR products were cut and recovered using the AxyPrepDNA gel recovery kit (AXYGEN, New York, USA), subsequently eluted by Tris_HCl [32]. The PCR products were quantified by the QuantiFluor™-ST Blue Fluorescence Quantification System (Promega, Madison, WI, USA). Miseq amplicon libraries were constructed and sequenced on the Illumina MiSeq-PE25 platform (Illumina, San Diego, CA, USA) in Majorbio Co. (Shanghai, China) [33]. The data were uploaded to the Majorbio Co., Cloud Platform (https://cloud.majorbio.com) for result analysis. All 16S rRNA sequence data can be downloaded from the National Center for Biotechnology Information (NCBI) under the project accession PRJNA1043446 (Submission ID: SUB13989547).

### 2.7. Hepatic Metabolomics Analysis

Livers of mice from each group (n = 4 replicates, three mouse livers were pooled as a replicate) were collected for untargeted metabolomics [34]. Approximately 50 mg of liver tissue was added to 200 μL of water homogenate and vortexed. 800 μL of methanol/acetonitrile (1:1) solution was vortexed for 60 s. After two rounds of low-temperature ultrasound (40 kHz) for 30 min, the proteins were precipitated by being placed in the refrigerator at ‒20 °C for 1 h. The samples were centrifuged at 4 °C and 14.000 r/min for 20 min, and the supernatants were freeze-dried and stored at ‒80 °C. The samples were analyzed using the UHPLC-ESI-Q-Exactive HF-X system (Thermo Fischer Scientific, Waltham, MA, USA). Chromatographic conditions and mass spectrometry conditions were detailed in Supplementary Text S1. Positive and negative ion chromatogram for all mixed samples (QC samples) are presented in Supplementary Figure S1. Raw data were pre-processed by Pareto scaling and statistically analyzed by orthogonal partial least squares discriminant analysis (OPLS-DA). The obtained metabolites were used for metabolite annotation and data processing using Progenesis QI (Waters, Milford, MA, USA) software. Metabolites with significant differences were screened and analyzed for metabolite interactions using the software MetaboAnalyst 6.0 with conditions VIP > 1 and *p* < 0.05 (http://www.metaboanalyst.ca/, accessed on 11 December 2023). Metabolic pathways were constructed based on KEGG enrichment analysis (http://www.genome.jp/kegg/pathway.html, accessed on 21 March 2024). The metabolomic data have all been uploaded to Metabolights (access number MTBLS8996).

### 2.8. Statistical Analysis

All data were analyzed using one-way analysis of variance (ANOVA) and by comparing mean differences between groups, with a *p*-value of <0.05 indicating significance. Statistical graphs were performed using GraphPad Prism 8.0 and values were shown as mean ± standard error (Mean ± SE), except for the metabolomics analysis. Annotated metabolites were performed using multivariate statistical analysis. A principal component analysis (PCA) was first performed to inspect the data variance and were generated with a cloud platform (Majorbio Co., Shanghai, China). Metabolic pathway analyses were generated with MetaboAnalyst 6.0 (https://www.metaboanalyst.ca/) [35]. The significance of individual metabolites between the four treatment groups was evaluated using ANOVA followed by Fisher’s post hoc analysis and a Holm FDR-correction, with a *p*-value of <0.05 indicating significance.

## 3. Results

### 3.1. Structural Characterization of HDPs

**Figure 1:**
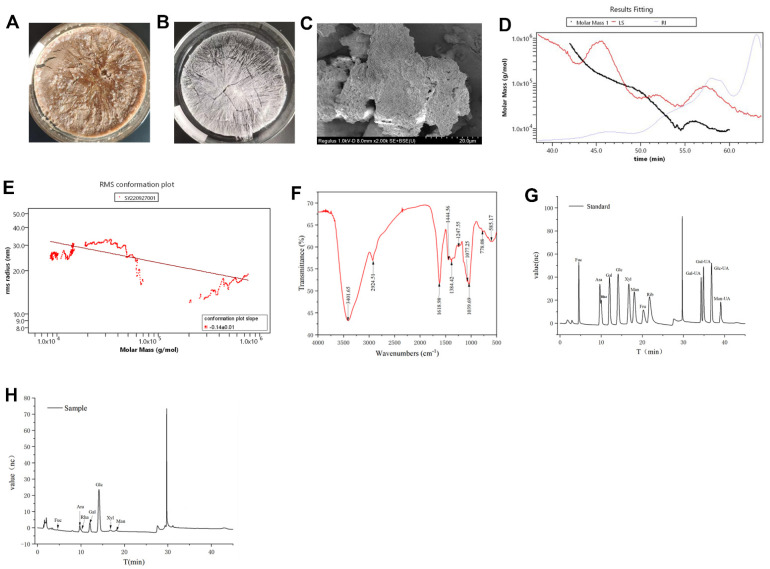
Structural characterization of *Hovenia dulcis* fruit peduncle polysaccharides (HDPs). (**A**) Crude HDPs picture, (**B**) HDPs picture, (**C**) scanning electron microscope image of HDPs, (**D**) molecular weight distribution plot of HDPs, (**E**) molecular conformation plot of HDPs, (**F**) FT-IR spectra of the HDPs, (**G**) ion chromatography spectra of monosaccharide standards, (**H**) ion chromatography spectra of HDPs.

The crude HDPs (Figure 1A) were purified to get the HDPs (Figure 1B), and the microstructure image of HDPs is shown in Figure 1C. The HDPs are aggregated clusters, stacked on top of each other, presenting a homogeneous porous structure, and the intermolecular morphology is tightly packed in terms of appearance and morphology, which is consistent with the molecular conformational map structure analysis (Figure 1E). The plot depicting the molecular weight distribution (Figure 1D) uses the assay’s retention time (Time, min) for the x-axis and the molar mass (g/mol) for the y-axis. Conversely, the molecular conformation plot (Figure 1E) reverses this, with molar mass (g/mol) on the x-axis and the root mean square radius (R.M.S. Radius, nm) on the y-axis. The Mn value (number average molecular weight) of HDPs was calculated as 13.163 kDa, Mw value (weight average molecular weight) as 29.73 kDa, Mz value (z average molecular weight) as 134.413 kDa, and Mp value (peak molecular weight) as 9.959 kDa. The slope of the graphs was −0.14 ± 0.01, and it can be concluded that the HDPs are small molecular weight polymers with compact and uniform spherical conformation.

As Figure 1F showed, the infrared spectrum of HDPs exhibits distinctive band characteristic of polysaccharides, highlighted by a pronounced absorption peak at 3401.65 cm^−1^, corresponding to the -OH stretching vibration signal peak, and another absorption peak at 2924.51 cm^−1^, which is attributed to the C-H stretching vibration signal peak. Both of these peaks are typical polysaccharide hydroxyl and alkyl groups, indicating the polysaccharide nature of the sample. A significant absorption peak appeared at 1618.98 cm^−1^, corresponding to the C=O stretching vibration and signifying the existence of -CHO groups, whereas the range of 1384.42 to1444.56 cm^−1^ represents the C-H variable angle vibration signal peaks, which are also characteristic infrared absorption peaks of polysaccharides. The presence of C-O-C and C-O-H bonds are indicated by the band around 1247.55 cm^−1^. The peaks at 1039.69 cm^−1^ and 1077.25 cm^−1^ are signal peaks generated by the stretching vibration of C-O and C-C bonds in the sugar ring, respectively, verifying that the HDPs contain pyranose monosaccharides. In addition, the analysis of the monosaccharide composition (Figure 1G,H and Supplementary Table S1) revealed that the HDPs were acidic polysaccharides and complex in structure. The major monosaccharide components consisted of fucose, rhamnose, arabinose, galactose, glucose, xylose, mannose, galacturonic acid, and glucuronic acid, with the following percentages (mol%): 0.55%, 11.41%, 5.15%, 14.15%, 60.66%, 2.48, 2.9%, 2.10%, and 0.62%, respectively. The content of glucuronic acid in the HDPs is very low, so its absorption peaks are not obvious in the infrared spectrogram.

### 3.2. Changes in Serum Lipid Levels and Liver Damage

**Figure 2:**
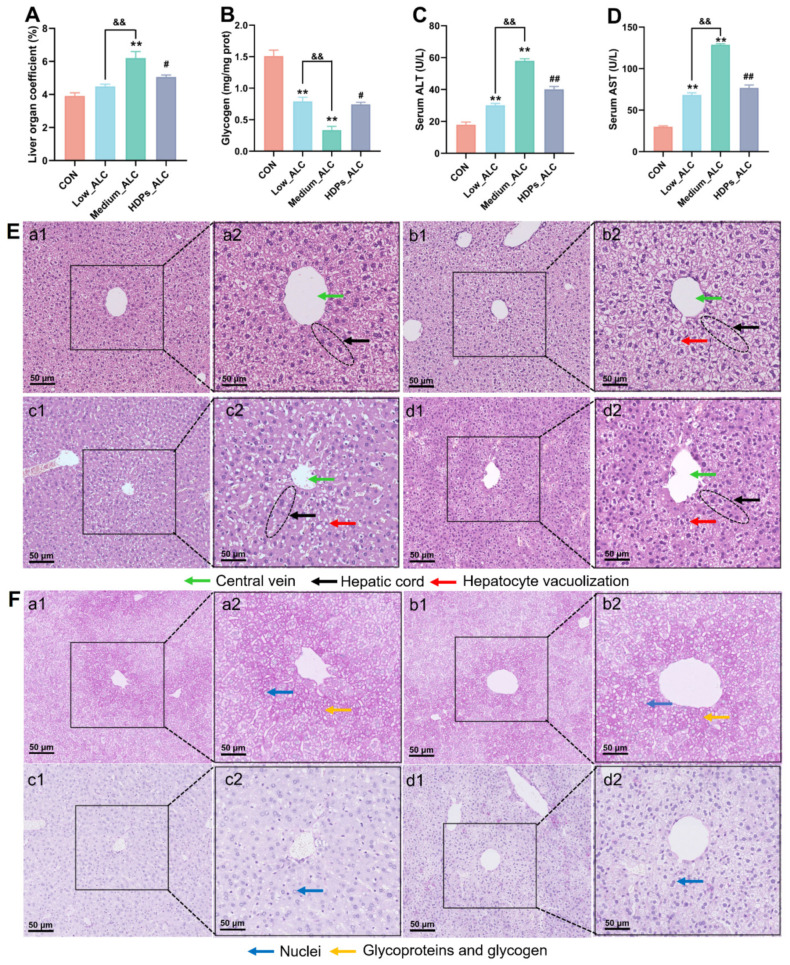
HDPs attenuate acute liver injury in alcohol-exposed mice. (**A**) Liver index, (**B**) hepatic glycogen, (**C**) serum ALT, (**D**) serum AST, (**E**) histological changes in HE-stained liver sections (bar = 50 μm), (**F**) histological changes in PAS-stained liver sections (Nuclei are colored blue, glycoproteins and glycogen are colored red, bar = 50 μm), **a**: CON, **b**: Low_ALC, **c**: Medium_ALC, **d**: HDPs_ALC. ** *p* < 0.01 compared with the CON group; && *p* < 0.01 compared with the Low_ALC group; # *p* < 0.05, ## *p* < 0.01 compared with the Medium_ALC group.

The levels of serum T-CHO, TG, LDL-C, and HDL-C were higher in the Low_ALC and Medium_ALC groups of mice than in the CON group (*p* < 0.05) (Figure S2). The levels of TG, TC, and LDL-C were significantly lower (*p* < 0.05) in the HDPs group compared with the Medium_ALC group (Figure S2A–C). Liver organ index, hepatic glycogen, serum ALT and AST activities were used as indicators of alcoholic acute liver function. Alcohol exposure significantly increased hepatic index, serum ALT, and AST activity, decreased hepatic glycogen content, and normalized after HDPs treatment compared to CON group (Figure 2A–D). Compared with the CON group, the Low_ALC and Medium_ALC groups had significant pathological changes, such as narrowing of the central vein (quantized in Supplementary Figure S3), vacuolization of hepatocytes and partial infiltration of inflammatory cells (Figure 2E), and reduction of hepatic glycogen (Figure 2F). Compared with the Medium_ALC group, the HDPs_ALC group did not show significant hepatocyte vacuolization and inflammatory cell infiltration (Figure 2E(d2)).

### 3.3. Changes in Hepatic and Intestinal Inflammatory Cytokines and Intestinal Enzyme Activities

**Figure 3:**
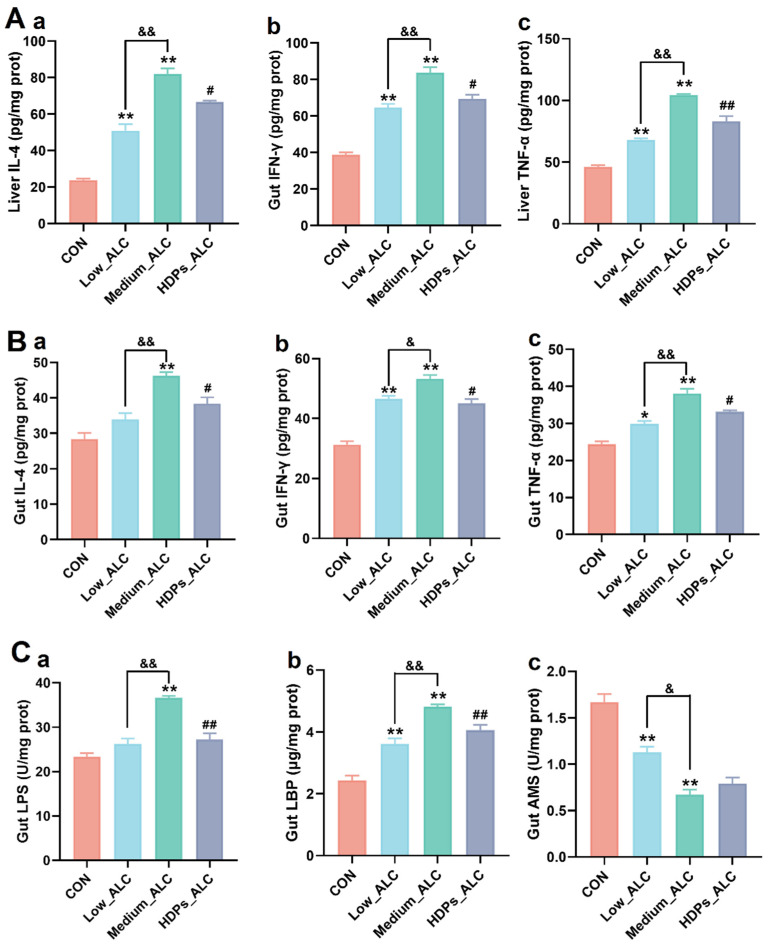
Effects of HDPs on hepatic and intestinal inflammatory cytokines and intestinal enzyme activities in alcohol-exposed mice. (**A**) Hepatitis cytokine changes, **a**: IL-4, **b**: IFN-γ, **c**: TNF-α; (**B**) intestinal inflammatory cytokine changes, **a**: IL-4, **b**: IFN-γ, **c**: TNF-α; (**C**) intestinal enzyme activity changes, **a**: LPS, **b**: LBP, **c**: AMS. Compared with the CON group, * *p* < 0.05, ** *p* < 0.01; compared with the Low _ALC group, & *p* < 0.05, && *p* < 0.01; compared with Medium_ALC group, # *p* < 0.05, ## *p* < 0.01.

**Figure 4:**
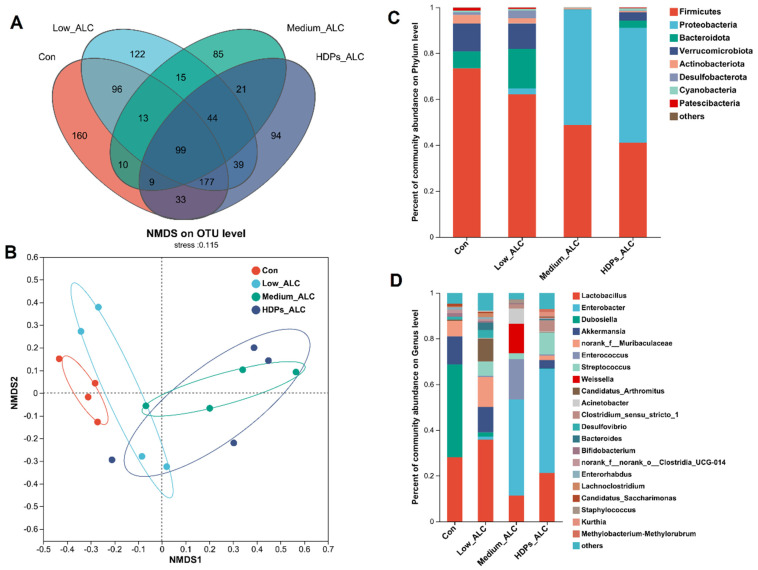
Differences in gut microbial community composition in alcohol-exposed mice. (**A**) Species Venn analysis, (**B**) NMDS (Nonmetric Multidimensional Scaling) analysis, (**C**,**D**) percentage of community abundance at the phylum or genus level.

Changes in pro-inflammatory cytokines (IL-4, IFN-γ and TNF-α) and intestinal enzyme activities in the liver and intestines of mice were quantified to assess the potential anti-inflammatory properties of HDPs. In the Low_ALC and Medium_ALC groups, the levels of these pro-inflammatory cytokines in both the liver and intestinal tissues were considerably elevated (*p* < 0.05) in comparison to the CON group. In the HDPs_ALC group, there was a significant decrease (*p* < 0.05) in the levels of these cytokines compared to the Medium_ALC group. (Figure 3A and Figure 4B). In contrast to the CON group, the Medium_ALC group exhibited a substantial increase (*p* < 0.05) in the concentrations of LPS and LBP within the intestinal tissue, along with a notable decrease (*p* < 0.05) in AMS levels. Conversely, when compared to the Medium_ALC group, the HDPs_ALC group showed a significant reduction (*p* < 0.05) in the levels of LPS and LBP (Figure 3C).

### 3.4. Dysbiosis of Gut Microbiota

Exposure to alcohol led to alterations in the diversity and composition of the gut microbiota of mice. The Sobs, Chao and ACE indices reflecting community richness were significantly lower in the alcohol-exposed group than in the CON group. The Shannon indices reflecting community diversity (1.55 ± 0.578) were significantly lower in the alcohol-exposed group than in the CON group (2.12 ± 0.675), while the Sobs, Chao, ACE and Shannon indices in the HDPs_ALC group were higher compared with those in the Medium_ALC group (Supplementary Table S2). Species analysis also revealed that the gut microbiota diversity of the alcohol-exposed group was reduced (Figure 4A). Structurally, the CON and Low_ALC groups were similar in community structure, and both were distributed in Quadrants I and II, while the Medium_ALC group was similar and distributed in Quadrants III and IV with the HDPs_ALC group. The CON group was clearly separated from the other treated groups, indicating a different community structure. The HDPs_ALC group showed a crossover with both Low_ALC and Medium_ALC groups, suggesting that the HDPs_ALC group microbiota structure was somewhere in between (Figure 4B). An examination of community composition revealed that at the phylum level, the Firmicutes phylum was markedly decreased in mice subjected to alcohol, while the Proteobacteria phylum exhibited a significant rise in the group receiving a high dosage of alcohol (Figure 4C). At the genus level, the *Enterobacter* genera significantly rose in the group given a high dose of alcohol. In contrast, the presence of *Dubosiella* was notably diminished, and the levels of *Lactobacillus* experienced a moderate decrease (Figure 4D).

### 3.5. Hepatic Metabolomics Changes

**Figure 5:**
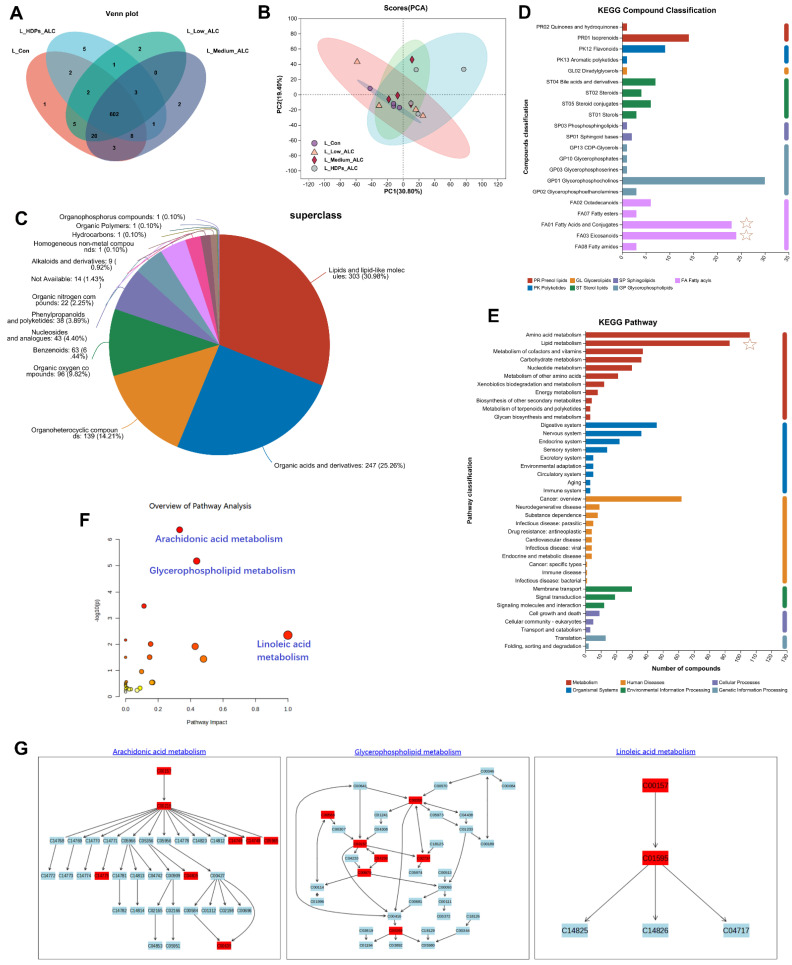
Changes in hepatic metabolomic profiles. (**A**) Metabolite Venn analysis; (**B**) PLS-DA analysis; (**C**) HMDB classification; (**D**) lipids KEGG classification; (**E**–**G**) KEGG pathway enrichment analysis.

To further understand the effects of HDPs on the metabolic response of the liver in alcohol-exposed mice, metabolic profiling of the liver was performed based on LC-MS in positive and negative ion mode. Venn, PCA and PLS-DA were employed to differentiate the hepatic metabolites among various treatment groups, aiming to identify potential biomarkers in the liver. Venn analysis highlighted variations in metabolite counts across the treatment groups, with the Medium_ALC group exhibiting a greater number of differential metabolites compared to the CON group (Figure 5A). The PCA analysis indicated that the CON group’s samples clustered closely together and were distinctly separated from the other groups (Supplementary Figure S4). PLS-DA was utilized to show the impact of HDPs on the metabolic profile. There was obvious clustering in the Low_ALC, Medium_ALC, and HDPs groups, both in positive and negative ion modes. There was a clear separation between the CON group and the alcohol-exposed group, suggesting that the liver metabolic profiles underwent obvious biochemical changes after alcohol exposure, whereas, the HDPs group showed a better clustering tendency, which may be because the HDPs exerted an intervention effect (Figure 5B). 

The HMDB classification of the identified metabolites showed that the different metabolites lipids led the different treatment groups up to 303 metabolites, which accounted for 30.98% of the metabolites (Figure 5C). The volcano plot of different significant metabolites showed variations in the levels of certain metabolites between the control and alcohol-exposed groups (Supplementary Figure S5). A selection of 285 differential metabolites, identified by their VIP values exceeding 1.0, were recognized as potential biomarkers with statistically significant differences between the groups (*p* < 0.05). In comparison to the control group, the Medium_ALC group exhibited a significant increase of 33 metabolites and a decrease of 25 metabolites. Conversely, the HDPs_ALC group showed a significant up-regulation of 24 metabolites and a down-regulation of 8 metabolites when compared to the Medium_ALC group. The majority of these significantly expressed metabolites fell into categories such as Fatty acyls (Fatty acids and conjugates, eicosanoids), glycerophospholipids, sterol lipids etc. (Figure 5D). KEGG-based metabolic analysis was performed to enrich the metabolic pathways of different hepatic metabolites in order to identify the important metabolic pathways affected. KEGG functional enrichment results were sequentially categorized as amino acid metabolism, lipid metabolism, metabolism of cofactors and vitamins, etc. (Figure 5E). Further analysis of lipid metabolism by applying MetaboAnalyst revealed that the major metabolic pathways altered in the HDPs_ALC group compared to the Medium_ALC group included arachidonic acid metabolism, glycerophospholipid metabolism, and linoleic acid metabolism, while also identifying two important metabolites—C00157 (phosphatidylcholine, a glycerophospholipid plays a central role in energy production) and C04230 (1-Acyl-sn-glycero-3-phosphocholine, a lysophospholipid involved in the breakdown of phospholipids) (KEGG compounds ID, Supplementary Table S3)—involved in the above metabolism (Figure 5F,G).

### 3.6. Correlations of Lipid Changes and Liver Metabolites with Gut Microbiota in Alcohol-Exposed Mice

#### 3.6.1. Relationship between Lipid Changes and Gut Microbiota

**Figure 6:**
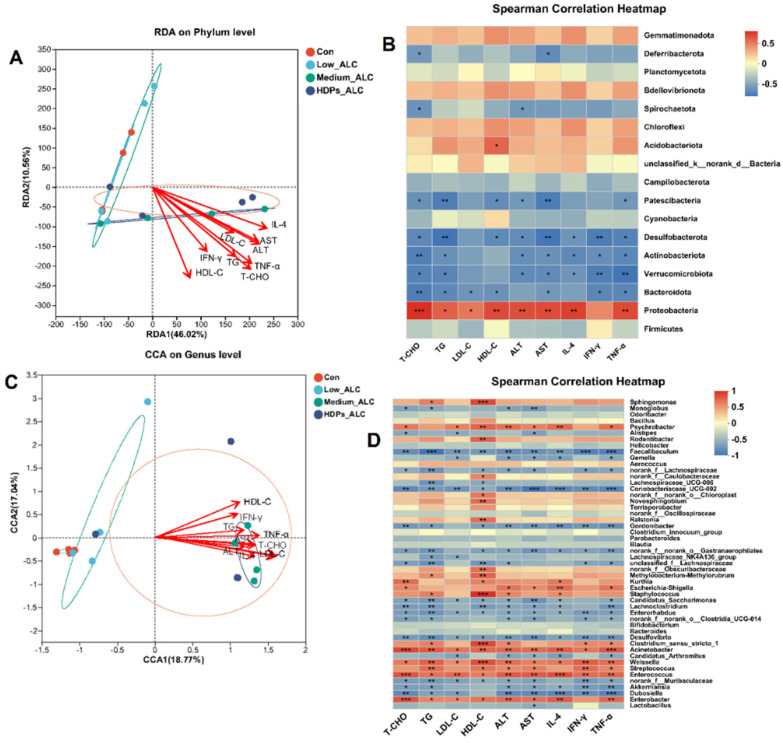
Spearman correlation analysis between the presence of phylum and genus in the gut microbiota and the corresponding levels of serum and liver markers. (**A**,**B**) The relationship between the relative quantities of certain phyla in the gut microbiota and parameters of serum and liver; (**C**,**D**) The correlation between the prevalence of specific genera in the gut microbiota and serum and liver parameters. Red arrows indicate serum and liver parameters (**A**,**C**); Positive (red) and negative (blue) correlations between genera and liver parameters (**B**,**D**), * *p* < 0.05, ** *p* < 0.01, ***p < 0.001.

RDA/CCA analysis and Spearman’s correlation heatmap demonstrated the correlation between alcohol-induced changes in gut microbiota composition and parameters such as serum biochemistry and hepatic inflammation. The phylum and genera in the Medium_ALC group and HDPs_ALC group showed significant positive correlation with lipid-related parameters (T-CHO, TG, LDL-C, HDL-C, ALT, AST, IL-4, IFN-γ, TNF-α) (Figure 6A,C). At the phylum level, Proteobacteria were significantly and positively correlated with T-CHO (r = 0.754), TG (r = 0.587), LDL-C (r = 0.503), HDL-C (r = 0.662), ALT (r = 0.653), AST (r = 0.658), and IL-4 (r = 0.697), TNF-α (r = 0.668), whereas Verrucomicrobiota, Desulfobacterota, Actinobacteriota, and Bacteroidota were significantly and negatively correlated with the above parameters (Figure 6B). At the genus level, *Enterobacter* and *Enterococcus* genera were significantly and positively correlated with those parameters, whereas *Dubosiella* and *Desulfovibrio* genera were significantly and negatively correlated with those parameters (Figure 6D).

#### 3.6.2. Relationship between Hepatic Metabolites and Gut Microbiota

**Figure 7:**
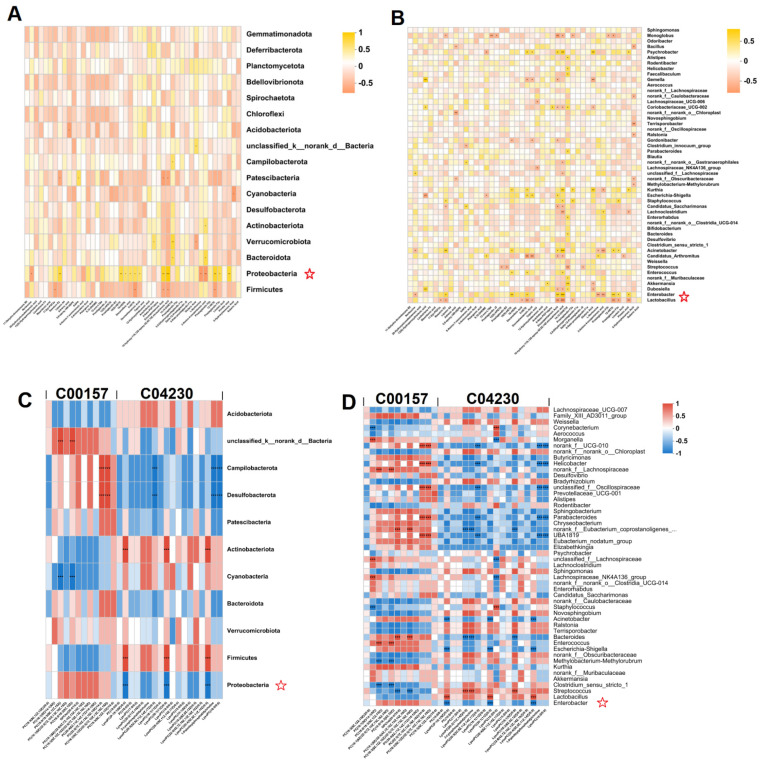
Positive and negative correlations of liver-identified differential metabolites with gut microbiota. (**A**,**B**) Spearman correlation heatmaps of intestinal flora in alcohol-exposed group with hepatic fatty acid metabolites at phylum and genera levels; (**C**,**D**) Spearman correlation heatmaps of intestinal flora in HDPs_ALC group at phylum and genera levels with liver-identified metabolic pathway C00157 and C04230 metabolites. Positive and negative correlations between genera and metabolites, * *p* < 0.05, ** *p* < 0.01, *** *p* < 0.001.

Spearman heatmaps visualized the relationship between gut flora and liver metabolism and revealed strong correlations between some genera and differential hepatic metabolites (cor > 0.5 or cor < 0.5). At the phylum level, there was a positive correlation between gut flora Proteobacteria and fatty acid metabolites in the liver in the alcohol-exposed group, and an increase in fatty acid metabolites in the liver was associated with an increase in the abundance of gut flora Proteobacteria (Figure 7A). At the genus level, there was a positive correlation between *Enterobacteria* genera and fatty acid metabolites of the liver in the alcohol-exposed group, and an increase in fatty acid metabolites of the liver was associated with a rise in the abundance of *Enterobacteria* genera, whereas there was a negative correlation between *Lactobacillus* genera and fatty acid metabolites of the liver (Figure 7B). After administration of HDPs treatment, C04230 metabolites in the HDPs_ALC group showed a significant decrease in Proteobacteria and a significant increase in Firmicutes at the phylum level (Figure 7C), and a significant decrease in *Enterobacter* abundance and a significant increase in *Lactobacillus* genera abundance at the genus level (Figure 7D).

## 4. Discussion

The intake of alcohol is recognized as a significant contributor to the development of liver disorders. *Hovenia dulcis* fruit peduncle’s primary naturally occurring active component, HDPs, holds promise in mitigating damage to the liver caused by alcohol. This investigation revealed that exposure to alcohol results in dyslipidemia, intensifies inflammation between the intestines and liver, disrupts the balance of gut microbiota, and impairs hepatic fatty acid metabolism, consequently triggering pathological alterations in liver tissue. The application of HDPs led to a notable reduction in the Proteobacteria phylum and a substantial enhancement in the Firmicutes phylum. It also increased the proportion of *Lactobacillus* and decreased that of *Enterobacteria*, exerting a regulatory influence on fatty acid metabolism. These findings imply that HDPs could be a valuable active component in addressing the reduction of gut microbial diversity and metabolic disruptions induced by alcohol consumption. 

### 4.1. HDPs Reduced Alcohol-Caused Lipid Abnormalities

More evidence has shown that alcohol consumption alters lipid metabolism [36,37]. Levels of TC, TG, HDL-C and LDL-C in the serum are key markers for the dysregulation of lipid metabolism. In this study, exposure to alcohol notably increased the concentrations of serum TC, TG, and LDL-C. This finding aligns with the previous study that reported a substantial rise in serum TC and TG levels in mice subjected to alcohol [38]. Nonetheless, after treatment with 100 mg/kg HDPs, the elevated TC and TG levels due to alcohol were brought back to levels closer to normal. Variations in the activities of ALT and AST in the serum serve as the most direct biochemical indicators of the severity of liver cell damage [39]. Additionally, alcoholic liver disease is marked by a pronounced rise in the liver index [40]. The findings indicate that therapy with HDPs significantly mitigated the increase in ALT and AST activities induced by alcohol consumption and normalized the liver index. By histological analysis of H&E, HDPs attenuated the destruction of liver lobules and the increase in hepatocyte volume after alcohol intake. Meanwhile, glycogen is the main form of energy stored in hepatocytes [41], and the alcohol-induced damaged hepatocytes in this study were unable to synthesize and store glycogen efficiently; therefore, the glycogen content was reduced. Histological results of PA-stained liver also showed that the HDPs_ALC group had normal glycogen in the liver tissues compared with the Medium_ALC group, which further confirmed the hepatoprotective effects of the HDPs. 

Within the group subjected to alcohol, there was a significant increase in levels of pro-inflammatory cytokines (IL-4, IFN-γ, TNF-α), indicating the role of inflammatory processes in the progression of initial alcohol-related liver damage. Nonetheless, the introduction of HDPs notably decreased the levels of these inflammatory cytokines. In line with this, the investigation demonstrated the *Hovenia dulcis Thumb* extracts’ capacity to counteract fat accumulation and inflammation in chronic alcohol-fed rats [23]. In addition, HDPs also reduced intestinal LPS and LBP levels and increased intestinal AMS activity. The above data fully indicate that HDPs seem to be effective in reducing pro-inflammatory cytokines, inhibiting intestinal inflammatory infiltration, and reducing the liver inflammation induced by ingested alcohol.

### 4.2. HDPs Alleviated Alcohol-Exposed Intestinal Dysbiosis and Hepatic Fatty Acid Metabolism Disorders

Excessive alcohol consumption disrupts the gut microbiome, resulting in dysbiosis, which is believed to be a key factor in the advancement of alcoholic liver disorders [42,43]. Furthermore, in the present study, HDPs were found to modulate the dysbiosis induced by alcohol intake. HDPs increased the Sobs, Chao, ACE and Shannon indices that were reduced after alcohol exposure, thereby increasing the abundance and diversity of gut microbial communities, which is important for maintaining gut health [44]. In the present study, it was found that after alcohol intake, Proteobacteria phylum increased and Firmicutes phylum decreased. This is consistent with the report that *Antrodin A* increased Firmicutes in alcohol-exposed mice [45]. Similarly, at the genus level, alcohol intake increased the relative abundance of *Enterobacter* and decreased the relative abundance of *Lactobacillus* and *Dubosiella*. Lactobacillus and *Dubosiella* are mainly found in the digestive tract of mammals, and they have a variety of positive effects on the host’s health, participating in the processes of food digestion and nutrient absorption, as well as in the absorption and digestion of nutrients, and they may also be involved in the regulation of the immune system and host metabolism [46]. Nevertheless, certain *Enterobacter* genera are one of the common infection pathogens with multi-drug resistance that can increase intestinal infections in the host. In this research, HDPs elevated the abundance of Firmicutes and *Lactobacillus*, thereby preserving the equilibrium of the gut microbial community. Moreover, the analysis of the relationship between the gut microbiota and markers of liver damage revealed that an upsurge in the Proteobacteria phylum and *Enterobacter* genera was directly linked to lipid levels and inflammatory markers. Conversely, a higher presence of the Firmicutes phylum and *Lactobacillus* genera was inversely associated with these lipids and inflammatory indicators. Consequently, HDPs had an important role in lipid metabolic homeostasis by regulating hepatic lipid metabolic pathways while maintaining the stability of intestinal flora composition. 

Serving as the pivotal organ for processing alcohol, the liver is the primary site susceptible to the harmful effects of alcohol [47]. It has a significant function in modulating the gut microbial community and their activities through multiple mechanisms, such as the generation of metabolites and enterohepatic cycling. Additionally, it reacts to the substances and nutrients from the intestines that are delivered via the portal circulation [44]. Prior investigations reveal that the intake of ethanol modifies the function of the intestinal barrier and the gut microbiota, resulting in an augmented release of endotoxins like LPS, which fosters significant communication between the liver and the intestine, worsening conditions of fatty liver, inflammation, and scarring in the liver [17]. Also, alcohol can cause alcoholic liver injury by inhibiting hepatic fatty acid metabolism and increasing hepatic lipid synthesis [9]. The hepatic metabolomics results of this study showed that alcohol exposure had the greatest negative influence on hepatic amino acid metabolism, lipid metabolism, and decreased fatty acid metabolic profiles leading to fatty acid metabolism disorders. The metabolic pathway enrichment analysis of hepatic metabolites in the HDPs_ALC group showed that supplementation with HDPs increased arachidonic acid metabolism, glycerophospholipid metabolism, and linoleic acid metabolism. The above metabolisms are important fatty acid metabolism processes in the organism. Arachidonic acid metabolism is involved in the regulation of cell growth, immune response, inflammation, and other physiological processes [48]. Glycerophospholipid metabolism involves a variety of metabolites such as phosphatidylinositol, triglycerides, phospholipids, etc., which are essential for maintaining cell membrane stability, signaling, cell proliferation and other functions [49]. Linoleic acid metabolism is mainly carried out through the triglyceride pathway and the cyclooxygenase pathway and is involved inflammatory response and other physiological processes [50]. It can be seen that HDPs can elevate alcohol-inhibited fatty acid metabolism and maintain lipid metabolism homeostasis by regulating these pathways.

In addition, the present study identified two key biomarkers linked to the metabolism of Arachidonic acid: the metabolites C00157 (phosphatidylcholine) and C04230 (1-Acyl-sn-glycero-3-phosphocholine), which play a role in this metabolic pathway. Furthermore, correlation analyses of intestinal flora and hepatic metabolites showed that after administration of HDPs treatment, C04230 metabolites in the HDPs_ALC group showed a significant reduction in Proteobacteria and a significant increase in Firmicutes at the phylum level, and a significant decrease in *Enterobacter* abundance and a marked elevation in *Lactobacillus* genera abundance at the genus level. These potential marker genera were most likely involved in the modulation of hepatic fatty acid metabolism disruptions mediated by HDPs.

### 4.3. Comparison of HDPs Studies

Through literature studies of HDPs and their analogs, we found that these studies focused on their antioxidant activity [13], potent immunostimulatory activity [51], effects on alcoholic liver injury and alcohol metabolism [18], as well as their roles in STZ-induced type 1 diabetes mellitus [21]. Most of the studies are structural analyses, and little information on mechanisms is available, except for the hypoglycemic mechanisms of HDPs-2A (a polysaccharide purified from H. dulcis) in T1DM rats revealed by Yang et al. [21]. For hepatoprotective activity in vivo, Wang et al. [52] suggested that HDPs provided considerable protection against liver damage caused by alcohol consumption by reducing oxidative stress. In contrast, from the perspective of gut microbes and hepatic metabolism, this study found that HDPs reduced alcohol-caused lipid abnormalities by alleviating alcohol-exposed intestinal dysbiosis and hepatic fatty acid metabolism disorders. Comparing our findings with other studies on HDPs, while most have focused on antioxidant activity and immunostimulatory effects, few have delved into the mechanisms of action. Our study provides novel insights into the hepato-protective activity of HDPs by addressing gut microbial and hepatic metabolic aspects.

## 5. Conclusions

**Figure 8:**
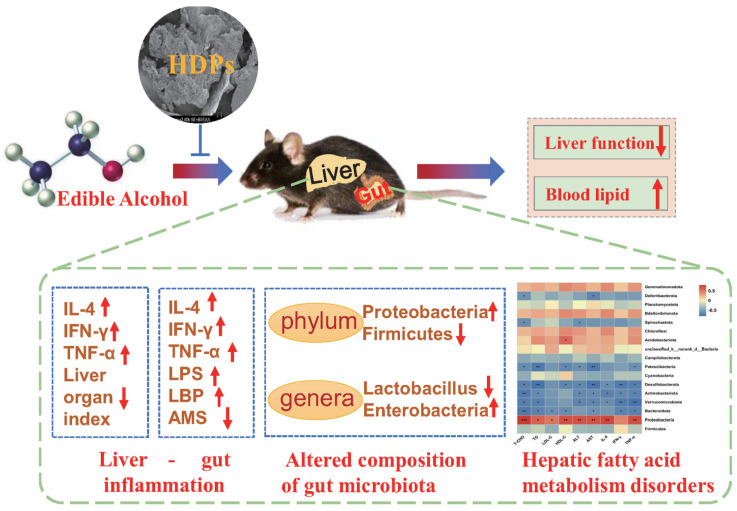
*Hovenia dulcis* fruit peduncle polysaccharides reduce intestinal dysbiosis and hepatic fatty acid metabolism disorders in alcohol-exposed mice. Positive (red) and negative (blue) correlations between genera and serum & liver parameters, * *p* < 0.05, ** *p* < 0.01, *** *p* < 0.001 Figure 8 was originally made by Liangyu Liu and Xudong Liu.

In the present study, HDPs were found to reduce dyslipidemia, decrease hepatic glycogen decline, and inhibit intestinal-hepatic inflammation; on the other hand, HDPs restored the composition of gut microbiota, and effectively regulated fatty acid metabolism disorders induced by alcohol intake, thus exerting protective effects against alcoholic liver injury. We further confirmed that the mechanism of HDPs may be related to the increase of intestinal *Lactobacillus* and decrease of *Enterobacteria*, and the increase of metabolites C00157 and C04230 involved in hepatic arachidonic acid metabolism, glycerophospholipid metabolism (Figure 8). This research offers valuable insights for the advancement of polysaccharides derived from natural extracts, like HDPs, and for the prevention of alcoholic liver disease.

Given that more studies have realized the potential health advantages and application significance of HDPs, there has been a transition from using unrefined extracts to more sophisticated, purified versions. In terms of microbial and nutritional research that focuses on HDPs in this study, our work will start with the analysis of the gut genera and metabolic pathways that have been unearthed, and further analyze the regulatory mechanisms that improve gut health. Overall, future research direction should be directed towards the comprehensive study of the active ingredients, pharmacological activities, clinical applications, biotechnological modifications, and nutritional effects of HDPs by utilizing an integrated and multidisciplinary approach from chemical, pharmacological, clinical, biotechnological, and nutritional perspectives, in order to further explore its prospective health and medicinal merits.

## Data Availability

The original contributions presented in the study are included in the article/Supplementary Material, further inquiries can be directed to the corresponding authors.

## Supplementary Materials

The following supporting information can be downloaded at: https://www.mdpi.com/article/10.3390/foods13081145/s1, Figure S1. The ion chromatogram; Figure S2. Effect of HDPs on serum lipid levels in alcohol-exposed mice; Figure S3: Central vein size quantification (μm) in the liver; Figure S4: Principal component analysis (PCA) score plots visualized the results from PCA discrimination analysis; Figure S5: Volcanic map of differential metabolites in positive ion mode; Table S1: The monosaccharide composition of the HDPs (mol%); Table S2: Alpha diversity analysis; Table S3: Identified metabolites C00157 and C04230 involved in arachidonic acid metabolism, glycerophospholipid metabolism, and linoleic acid metabolism.

## Abbreviations

Alanine aminotransferase	ALT
Aspartate aminotransferase	AST
Control	CON
Fourier transform infrared	FT-IR
High-density lipoprotein cholesterol	HDL-C
*Hovenia dulcis* fruit peduncle polysaccharides	HDPs
HDPs + Medium dose of alcohol	HDPs_ALC
Interferon-Gamma	IFN-γ
Low-density lipoprotein cholesterol	LDL-C
Low dose of alcohol	Low_ALC
Medium dose of alcohol	Medium_ALC
National center for biotechnology information	NCBI
Orthogonal partial least squares discriminant analysis	OPLS-DA
Principal component analysis	PCA
Triglycerides	TG
Total cholesterol	T-CHO
Tumor Necrosis Factor-Alpha	TNF-α
Ultra-High Performance Liquid Chromatography	UHPLC
World Health Organization	WHO
