# Supplementary material for: Hovenia dulcis Fruit Peduncle Polysaccharides Reduce Intestinal Dysbiosis and Hepatic Fatty Acid Metabolism Disorders in Alcohol-Exposed Mice
Source: Foods. 2024 Apr 9;13(8):1145. doi: 10.3390/foods13081145 (PMC11049514; doi:10.3390/foods13081145)
Supplement: Supplementary file 1 [file foods-13-01145-s001.zip › foods-2852713-supplementary.pdf]

Supplementary Material

1 Supplementary Figures

1.1 Figure S1

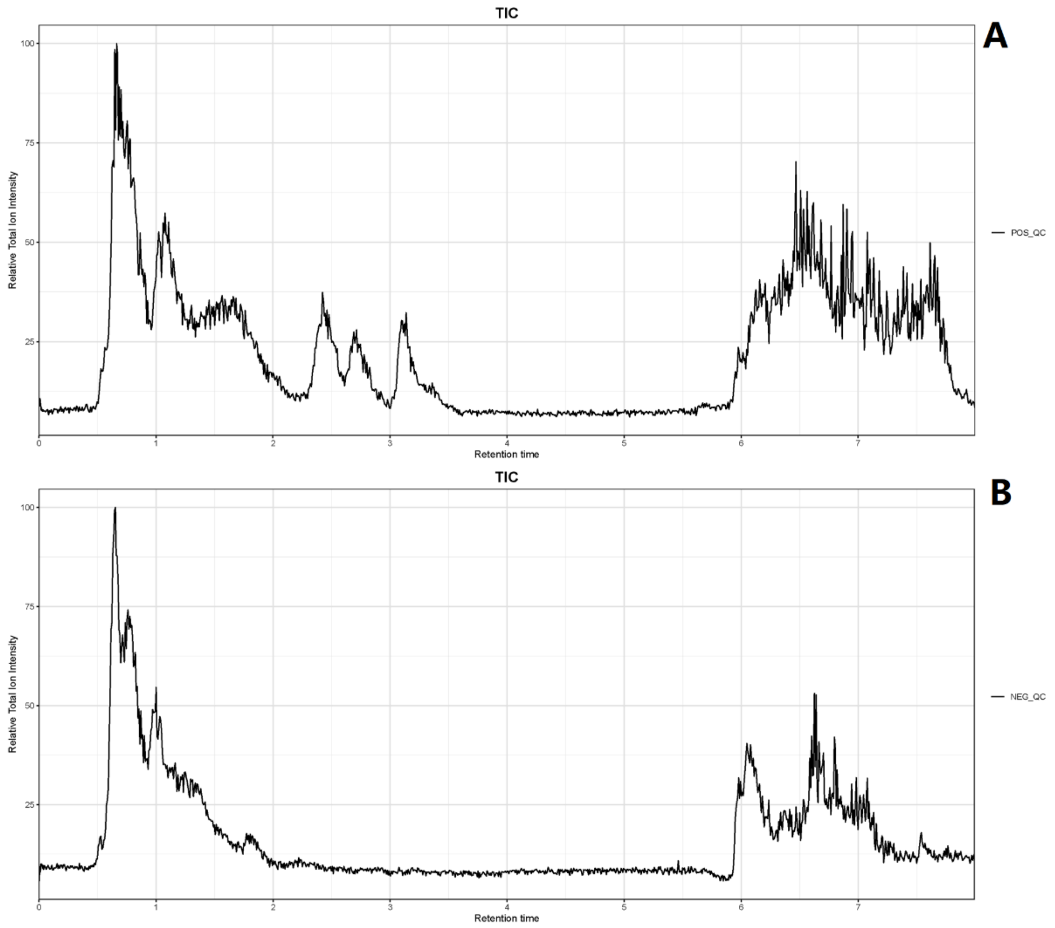

Figure S1. The ion chromatogram in quality control samples. (A) Positive model; (B) Negative model.

1.2 Figure S2

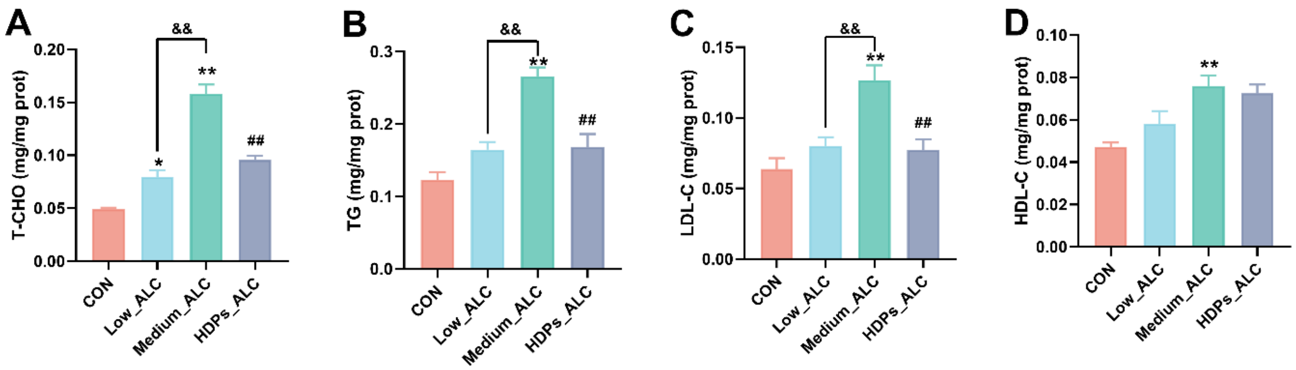

Figure S2. Effect of HDPs on serum lipid levels in alcohol-exposed mice. (A) serum T-CHO; (B) serum TG; (C) serum LDL-C; (D) serum HDL-C. *p < 0.05, **p < 0.01 compared with the CON group; &&p < 0.01 compared with the Low_ALC group; ##p < 0.01 compared with the Medium_ALC group.

1.3 Figure S3

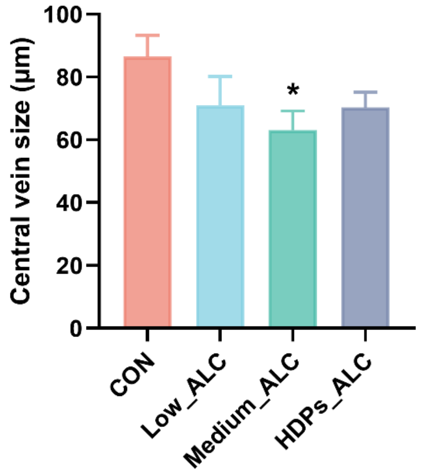

Figure S3. Central vein size quantification (μm) in the liver. At least 5 central veins were randomly selected from pathologic tissue scan sections of the liver, and their sizes were measured by NanoZoomer Digital Pathology software to obtain the mean values. Compared with the CON group, *$p < 0.05$.

1.4 Figure S4

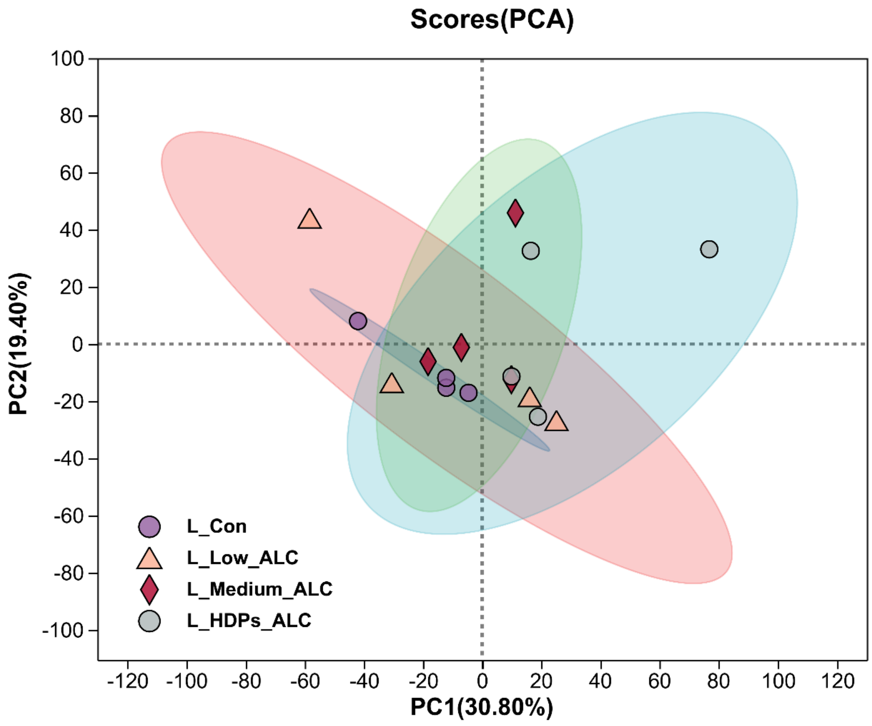

Figure S4. Principal component analysis (PCA) score plots visualized the results from PCA discrimination analysis. A confidence ellipse indicates that the "true" samples in this group are distributed within this region at the 95% confidence level; beyond this region, the samples may be anomalous.

1.5 Figure S5

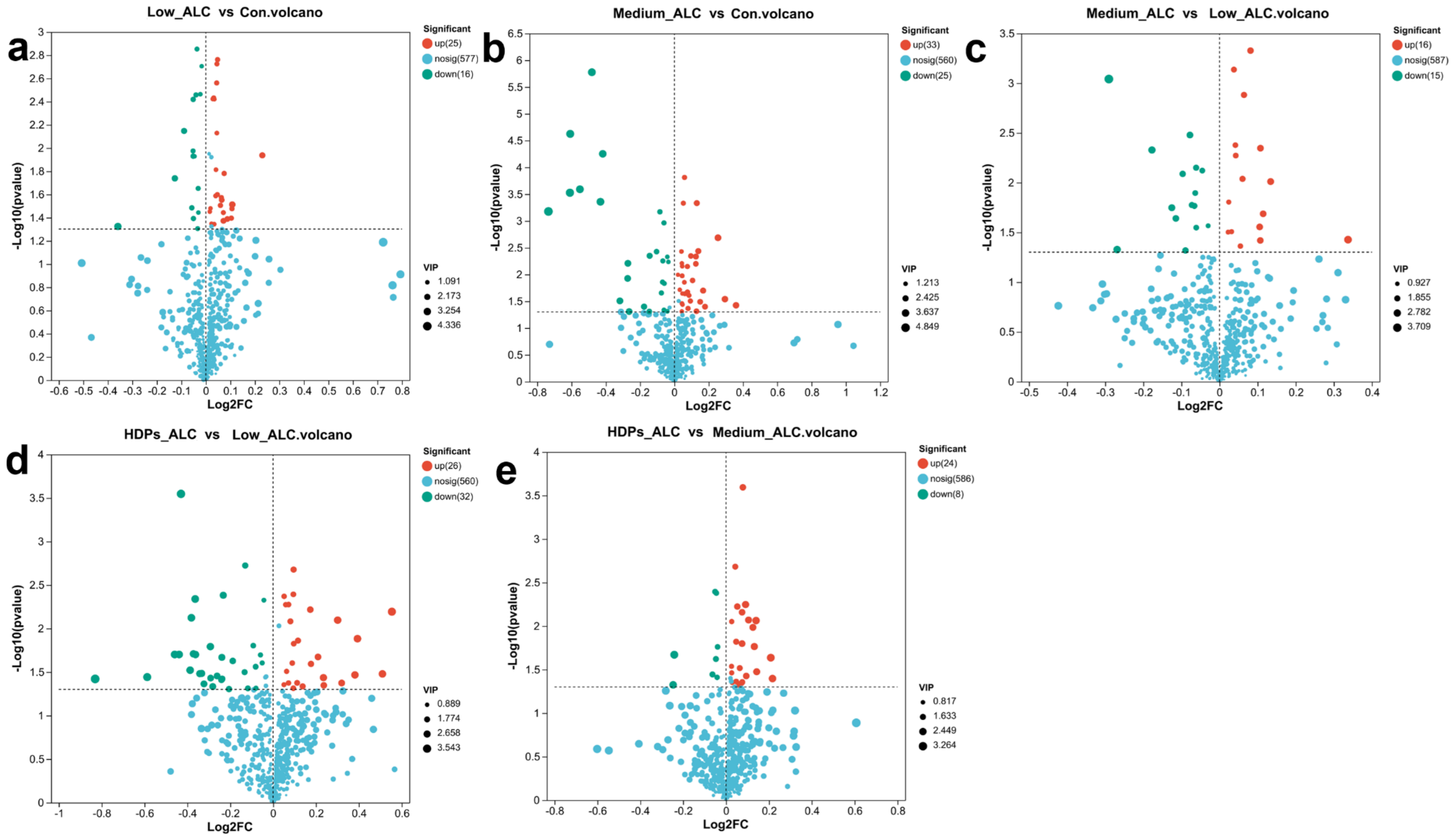

Figure S5. Volcanic map of differential metabolites in positive ion mode. a: Low_ALC vs CON (up 25, down 16), b: Medium_ALC vs CON (up 33, down 25), c: Medium_ALC vs Low_ALC (up 16, down 15), d: HDPs_ALC vs Low_ALC (up 26, down 32), e: HDPs_ALC vs Medium_ALC (up 24, down 8).

2 Supplementary Tables

2.1 Table S1

Table S1. The monosaccharide composition of the HDPs (mol%)

Fuc	Ara	Rha	Gal	Glc	Xyl	Man	Gal-UA	Glc-UA
0.55	11.41	5.15	14.15	60.66	2.48	2.90	2.10	0.62

The monosaccharide composition of HDPs by high performance anion exchange chromatography (HPAEC) is shown in Table S1. The results showed that the HDPs were acidic polysaccharides and complex in structure, and their major monosaccharide components consisted of fucose, rhamnose, arabinose, galactose, glucose, Xylose, mannose, galacturonic acid, and glucuronic acid, with the following percentages (mol%): 0.55%, 11.41%, 5.15%, 14.15%, 60.66%, 2.48, 2.9%, 2.10%, and 0.62%, respectively.

2.2 Table S2

Table S2. Alpha diversity analysis (n = 4).

Estimators	Con	Low_ALC	Medium_ALC	HDPs_ALC
ace	376.8 ± 128.1	249.6 ± 126.3	137.0 ± 115.4*	242.7 ± 101.8
chao	357.6 ± 126.5	250.6 ± 124.3	126.5 ± 116.4*	236.9 ± 98.3
shannon	2.12 ± 0.675	2.11 ± 0.675	1.55 ± 0.578*	1.69 ± 0.131
sobs	297.5 ± 122.5	221.3 ± 122.5	108.8 ± 112.4*	215.8 ± 85.5

Compared with the CON group, *$p < 0.05$.

2.3 Table S3

Table S3. Identified metabolites C00157 and C04230 involved in arachidonic acid metabolism, glycerophospholipid metabolism, and linoleic acid metabolism.

KEGG Compound ID	Metabolite	Metab ID	Formula	Retention time	HMDB Class	M/Z
C00157	PC(18:3(9Z,12Z,15Z)/20:0)	metab_541	C46H86NO8P	7.21	Glycerophospholipids	834.60
C00157	PC(18:1(9Z)/14:1(9Z))	metab_1029	C40H76NO8P	6.34	Glycerophospholipids	730.54
C00157	PC(18:0/18:3(9Z,12Z,15Z))	metab_1220	C44H82NO8P	6.99	Glycerophospholipids	784.58
C00157	PC(16:1(9Z)/22:5(7Z,10Z,13Z,16Z,19Z))	metab_1274	C46H80NO8P	7.31	Glycerophospholipids	806.57
C00157	PC(16:0/18:2(9Z,12Z))	metab_1730	C42H80NO8P	7.19	Glycerophospholipids	758.57
C00157	GPCho(20:4/16:0)	metab_1781	C44H80NO8P	6.99	Glycerophospholipids	804.55
C00157	PC(18:1(9Z)/22:6(4Z,7Z,10Z,13Z,16Z,19Z))	metab_1992	C48H82NO8P	6.44	Glycerophospholipids	832.58
C00157	PC(18:3(9Z,12Z,15Z)/22:5(7Z,10Z,13Z,16Z,19Z))	metab_4046	C48H80NO8P	7.00	Glycerophospholipids	830.57
C00157	PC(20:4(8Z,11Z,14Z,17Z)/16:1(9Z))	metab_4060	C44H78NO8P	7.21	Glycerophospholipids	780.55
C00157	PC(22:5(7Z,10Z,13Z,16Z,19Z)/14:0)	metab_4078	C44H78NO8P	7.51	Glycerophospholipids	780.55
C00157	PC(18:2(9Z,12Z)/22:5(7Z,10Z,13Z,16Z,19Z))	metab_4093	C48H82NO8P	7.66	Glycerophospholipids	832.58
C00157	PC(18:3(9Z,12Z,15Z)/16:0)	metab_4142	C42H78NO8P	7.61	Glycerophospholipids	756.55
C04230	PC(18:0/0:0)	metab_575	C26H54NO7P	7.62	Glycerophospholipids	524.37
C04230	LysoPC(P-18:1(9Z)/0:0)	metab_1367	C26H52NO6P	7.71	Glycerophospholipids	538.39
C04230	PC(16:0/0:0)	metab_1591	C24H50NO7P	7.65	Glycerophospholipids	518.32
C04230	LysoPC(17:0/0:0)	metab_1740	C25H52NO7P	7.16	Glycerophospholipids	510.35
C04230	LysoPC(20:2(11Z,14Z)/0:0)	metab_1782	C28H54NO7P	6.99	Glycerophospholipids	548.37
C04230	2-Lysophosphatidylcholine	metab_1886	C26H54NO7P	6.67	Glycerophospholipids	546.35
C04230	LysoPC(20:5(5Z,8Z,11Z,14Z,17Z)/0:0)	metab_1994	C28H48NO7P	6.44	Glycerophospholipids	542.32
C04230	LysoPC(18:1(11Z)/0:0)	metab_4036	C26H52NO7P	6.89	Glycerophospholipids	522.35
C04230	LysoPC(20:1(11Z)/0:0)	metab_4086	C28H56NO7P	7.62	Glycerophospholipids	550.39
C04230	LysoPC(20:4(8Z,11Z,14Z,17Z)/0:0)	metab_5285	C28H50NO7P	7.55	Glycerophospholipids	588.33
C04230	LysoPC(15:0/0:0)	metab_5297	C23H48NO7P	7.54	Glycerophospholipids	480.31
C04230	LysoPC(16:1(9Z)/0:0)	metab_5622	C24H48NO7P	6.42	Glycerophospholipids	538.32
C04230	LysoPC(18:3(6Z,9Z,12Z)/0:0)	metab_5675	C26H48NO7P	6.36	Glycerophospholipids	562.31
C04230	LysoPC(22:6(4Z,7Z,10Z,13Z,16Z,19Z)/0:0)	metab_6583	C30H50NO7P	6.37	Glycerophospholipids	612.33
C04230	LysoPC(20:4(5Z,8Z,11Z,14Z)/0:0)	metab_6672	C28H50NO7P	6.41	Glycerophospholipids	588.33

C04230	1-Palmitoylphosphatidylcholine	metab_6816	C24H50NO7P	6.67	Glycerophospholipids	540.33
C04230	LysoPC(16:0/0:0)	metab_6867	C24H50NO7P	7.54	Glycerophospholipids	540.33

3 Supplementary Texts

3.1 Text S1 Chromatographic conditions and mass spectrometry conditions.

Chromatographic conditions: the column was ACQUITY UPLC HSS T3 (100 mm × 2.1 mm, 1.8 μm; Waters, Milford, USA); mobile phase A was 95% water + 5% acetonitrile (containing 0.1% formic acid), and mobile phase B was 47.5% acetonitrile + 47.5% isopropanol + 5% water (containing 0.1% formic acid); the injection volume was 3 μL, and the column temperature was 40 °C. **Mass spectrometry conditions:** the sample is ionized by electrospray ionization, and the mass spectrometry signals are collected in positive and negative ion scanning modes, respectively.
